# Percutaneous Placement of Iliosacral Screws Under the Guidance of Axial View Projection of the S1 Pedicle: a Case Series

**DOI:** 10.1038/s41598-017-08262-w

**Published:** 2017-08-11

**Authors:** Yingchao Yin, Zhiyong Hou, Ruipeng Zhang, Lin Jin, Wei Chen, Yingze Zhang

**Affiliations:** Department of Orthopaedic Surgery, Third Hospital of Hebei Medical University, Shijiazhuang, Hebei Province China

## Abstract

The aim of this study was to evaluate the safety and efficacy of percutaneous placement of iliosacral screws under the guidance of axial view projection of the S1 pedicle clinically. This case series includes 58 consecutive unstable pelvic injury patients, which were treated with iliosacral screws between July 2011 and July 2016. Patients were divided into two groups: normal sacrum (*n* = 31) and dysmorphic sacrum (*n* = 27). A single orthopedic surgeon operated on all patients, with percutaneous placement of iliosacral screws under the guidance of axial view projection of the S1 pedicle. The time needed for screw insertion and the radiation exposure time were recorded. Chi-squared test and Student *t*-test were used to analyze the differences between the two groups. Sacral dysmorphism was present in 47% of patients. The median time for screw insertion and radiation exposure time in these two groups showed no statistical difference (*P* > 0.05). No clinical complications or malpositioned screws occurred in any case. Preoperative pelvic CT is necessary to determine the sacral osseous anatomy. In patients with either a normal or dysmorphic sacrum, iliosacral screws can be placed by this method with less radiation exposure and complications than in the conventional method.

## Introduction

Sacroiliac dislocation and sacral fracture, which usually accompanies disruption of the pelvic rami or fractures of the symphysis pubis, is the most unstable type of pelvic ring injury. Sacroiliac dislocations and sacral fractures are traditionally stabilized using iliosacral screw fixation. The partially-threaded screws pass through the external surface of the posterior iliac wing and into the body of the first sacral vertebra (S1) to achieve sacroiliac joint compression and fixation. Percutaneous insertion of iliosacral screws is an attractive procedure with the well-known advantage of providing excellent biomechanical stability for posterior ring injury through a minimally invasive approach^1, 2^.

The conventional method used to accomplish the percutaneous insertion of iliosacral screws derives mainly from the technique described by Matta and Saucedo^1^ and Routt *et al*.^3^, in which C-arm fluoroscopy is used to visualize the pelvis in three views: inlet, outlet and true lateral views. However, the procedure has a long learning curve preoperatively and is technically demanding intraoperatively^4^. Surgeons have to withstand substantial radiation exposure and spend considerable time adjusting the three views during the procedure^5, 6^. It has recently been reported that sacral anatomic variations exist in nearly half of the adult population^7, 8^. The dysmorphic sacrum has an oblique osseous pathway that results in inability to clearly determine the safety zone for screw placement on true lateral view, which makes the conventional projection method of iliosacral screw placement difficult or impossible. Additionally, poor fluoroscopic image quality on the fluoroscopic monitor can result in complications such as malpositioned screws or injury to adjacent neurovascular structures. Malpositioned screw placement reportedly occurs in 2–15% of cases^3, 9, 10^.

To position the iliosacral screws speedily and efficiently, Hou *et al*. described a new technique of iliosacral screw placement with C-arm fluoroscopy visualizing the pedicle view of S1, which was shown to be efficient and safe in cadavers^11^. The present investigation was designed to prove that the sacral pedicle axial view projection is an optimal and practical radiographic technique for percutaneous placement of iliosacral screws clinically.

## Materials and Methods

The study was approved by the Regional Ethics Board of Hebei Medical University, Shijiazhuang, China, and the institutional guidelines for the care and treatment of patients were rigorously followed. Informed consent was obtained from all individual participants included in the study.

### Patients

**Figure 1:**
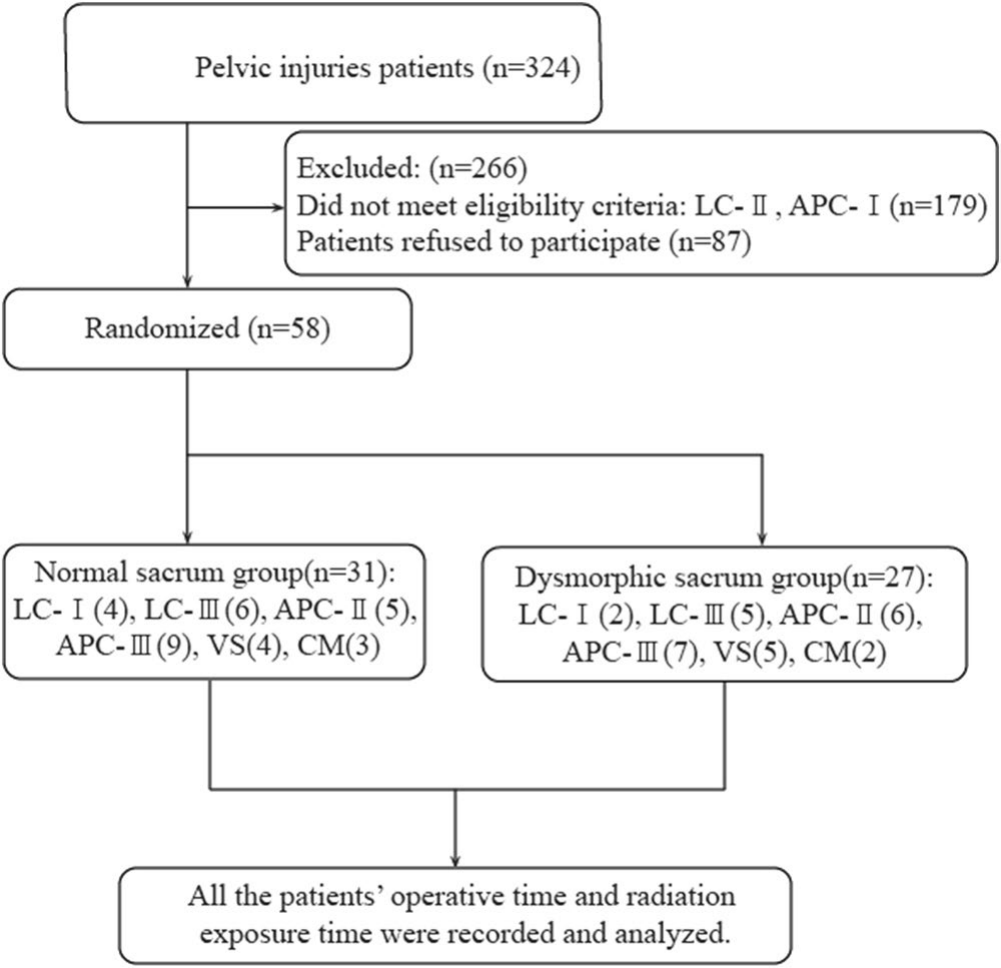
Recruitment of patients with pelvic injuries and data collection schedule.

**Figure 2:**
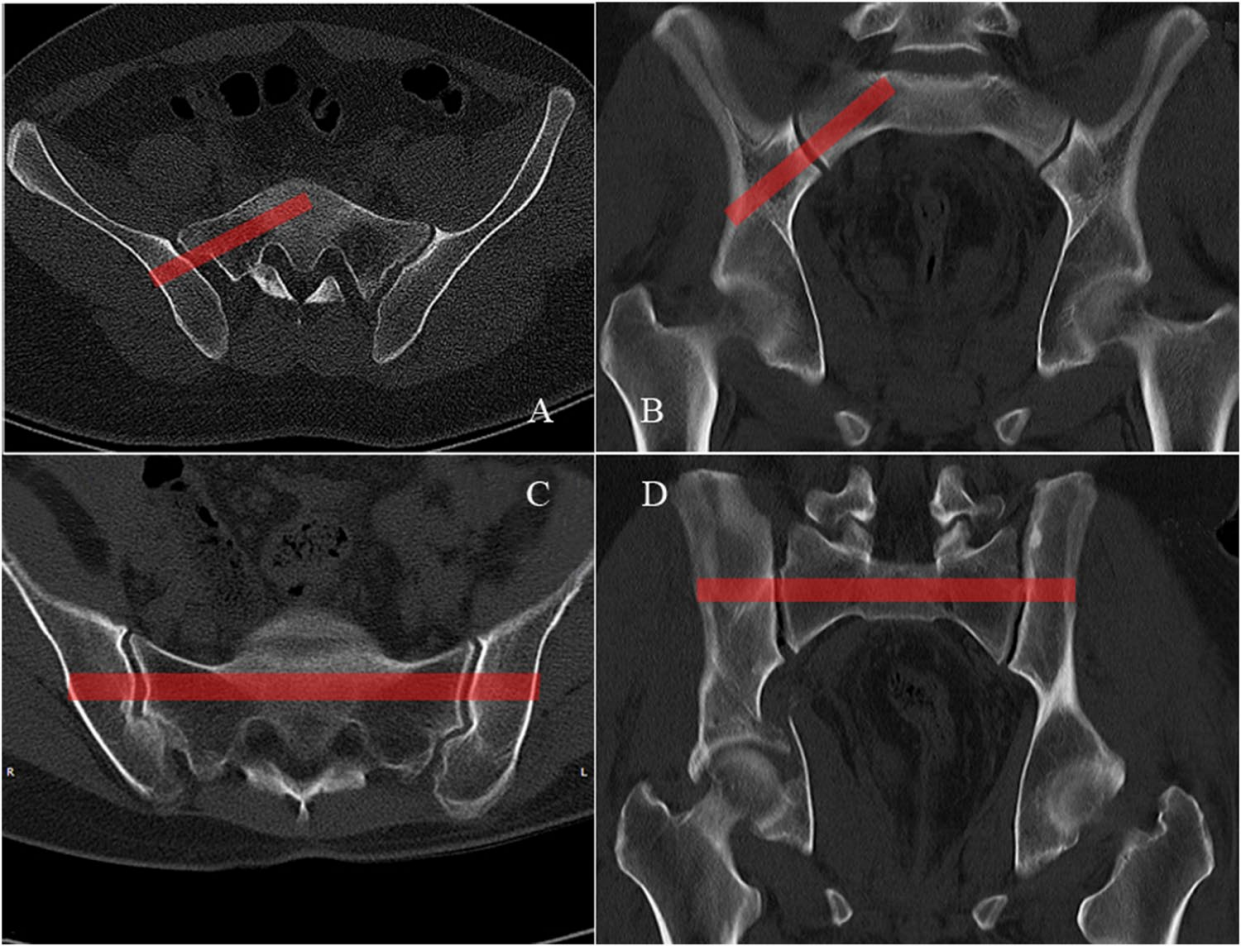
(**A,B**) The top sacral segment axial CT imagery of a patient with sacral dysmorphism. The dysmorphic sacrum has an oblique osseous pathway, which only allows oblique screw placement. (**C,D**) The top sacral segment axial CT imagery of a patient with a normal sacrum. The red column represents the pathway available for transverse screw placement.

Between July 2011 and July 2016, 324 patients with posterior pelvic ring injuries were treated with percutaneous placement of iliosacral screws at our department (a single, level 1 trauma center). In all patients, the pelvis was evaluated on preoperative radiography (including anteroposterior, inlet, and outlet views) and CT. Two experienced orthopedists evaluated the imaging data and classified each fracture using the Young-Burgess system^12^. Inclusion criteria were type LC-I, LC-III, APC-II, APC-III, VS and CM fractures (Fig. 1), which were identified with posterior pelvic ring injuries and treated by iliosacral screw placement. Patients were divided into the normal sacrum group and the dysmorphic sacrum group (Fig. 2) according to preoperative evaluation based on the criteria described by Routt *et al*.^3^. All patients were hemodynamically stable at the time of the indicatory procedure, and percutaneous placement of iliosacral screws was chosen for posterior ring fixation. A single orthopedic surgeon (HZY) operated on all patients.

The medical records including inpatient medical records, operative notes, and postoperative radiographs for each patient were reviewed. In particular, the operation time from initial preparation to completion of the first screw insertion and associated radiation exposure times were recorded.

All available postoperative radiographs (the pelvis anteroposterior, inlet, outlet views) were evaluated by the investigators, excluding the operating surgeon. The inlet view was used to measure screw placement about the anterior vertebral body and the posterior sacral canal; the outlet view was used to evaluate screw placement in relation to the adjacent sacral foramina. The screw was rated as malpositioned if any view showed signs of intrusion of the bone according to the criteria of Van den Bosch *et al*.^13^.

### Technique

**Figure 3:**
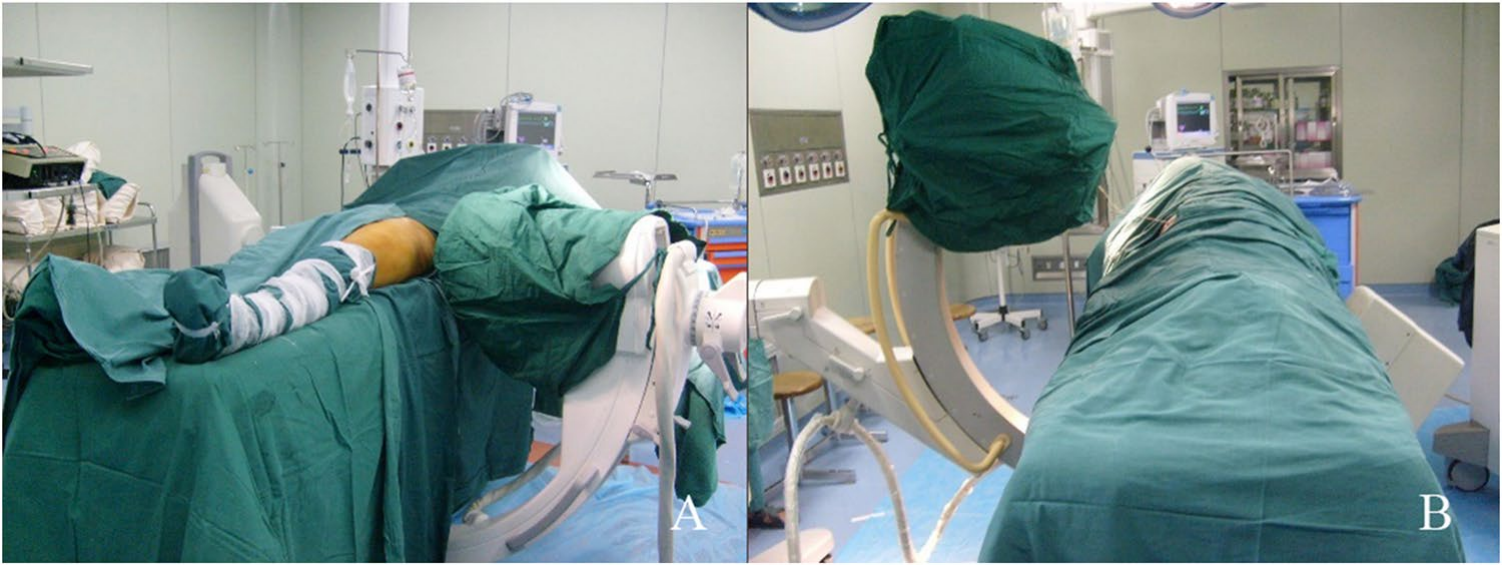
(**A**) The C-arm fluoroscope unit was positioned for the lateral view of the sacrum. (**B**) The angle of the C-arm was fixed to make it project along the axial view of the pedicle of the first sacral vertebra.

The technique of percutaneous iliosacral screw placement under the guidance of axial view projection of the S1 pedicle has been described in detail^11^. A general or epidural anesthesia was used, and the patient was place in prone or supine position on the radiolucent operating table (Fig. 3A,B).

**Figure 4:**
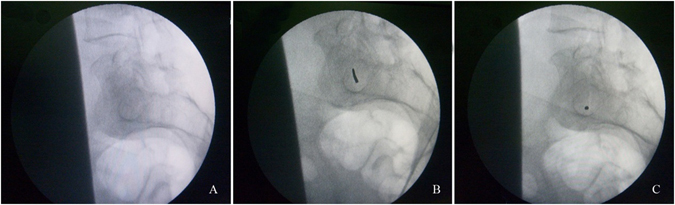
(**A**) A clear oval track image appeared on the fluoroscopy. (**B**) The starting point of the guide pin was located in the center of the oval track. (**C**) The projection of the guide pin became a point inside the oval track.

The anterior ring of pelvic disruption was reduced anatomically and fixed by an external fixator or by plates using the Stoppa approach^14^. The posterior pelvic ring dislocation was reduced automatically or by manual manipulation. The C-arm fluoroscope unit was positioned so that the lateral view of the sacrum illustrated clearly the outline of the body of S1. The C-arm fluoroscope tube was then gradually changed to ventral and cephalad. The image of the body of S1 was observed during the changing of the angle of the C-arm projection. When the bright oval track image appeared (Fig. 4A), which is the axial view of the pedicle of S1, the C-arm was fixed (Fig. 3B).

The oval track reveals the medullary pathway for percutaneous screw fixation of the sacroiliac joint of S1. Under the guidance of the C-arm fixed in the sacral pedicle axial view, the starting point of the guide pin was located in the center of the oval track (Fig. 4B) and the orientation was adjusted. When the guide pin was parallel to the direction of the X-ray projection, the projection of the guide pin became a point inside the oval track (Fig. 4C).

**Figure 5:**
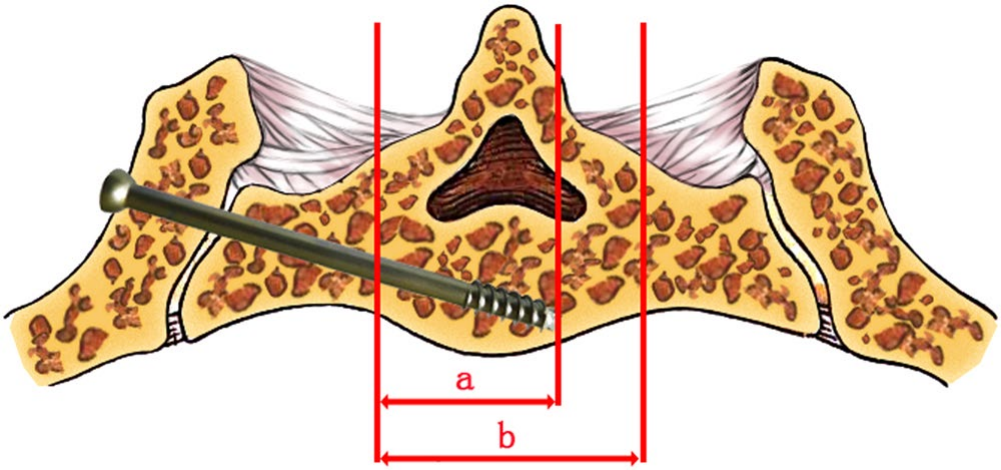
The depth of the cannulated lag screw (a), which is inserted into the body of the first sacral vertebra, should be no more than 2/3 of the vertebral body (b).

The direction of the guide pin was maintained, and the pin was inserted a few centimeters using battery-powered equipment or a hammer. Using the anteroposterior view, the guide pin was advanced into the S1 body. The length of the guide pin in the bone was measured. The screw path was drilled and tapped in order. A 6.5 cannulated screw of appropriate length was then inserted over the guide wire. Generally, the depth of the cannulated lag screw was no more than two-thirds of the transverse diameter of the vertebral body in the anteroposterior view (Fig. 5).

### Statistical analysis

Continuous data were presented as the mean and standard deviation. The distributions of all variables were evaluated for normality using a combination of quantile-quantile (Q-Q) plots, and Shapiro-Wilk tests. The difference in percentage of sex and fracture pattern between the two groups was determined using the Chi-squared test. The Student *t*-test was used to analyze the differences between groups in mean age, operation time and radiation exposure time. All statistical analyses were performed using IBM SPSS Statistics for Windows, version 21.0 (IBM, Armonk, NY, USA). A value of *P* less than 0.05 was considered significant.

## Results

Demographic characteristics of the patients are summarized in Table 1. There were 27 patients (47%) diagnosed with dysmorphic sacrum on axial CT scans. The normal sacrum group included 18 males and 13 females with a median age of 37.8 ± 9.9 years (range, 22–60 years); the pelvic injury types were LC-I (*n = *4), LC-III (*n = *6), APC-II (*n = *5), APC-III (*n = *9), VS (*n = *4) and CM (*n = *3). The dysmorphic sacrum group included 15 males and 12 females with a median age of 42.4 ± 10.5 years (range, 25–61 years); the pelvic injury types were LC-I (*n = *2), LC-III (*n = *5), APC-II (*n = *6), APC-III (*n = *7), VS (*n = *5) and CM (*n = *2).

**Table 1:** Demographic characteristics.

Parameter	Normal sacrum group(n = 31)	Dysmorphic sacrum group(n = 27)	*P*
Gender, number (%)			0.847
Male	18(58.1%)	15(55.6%)	
Female	13(41.9%)	12(44.4%)	
Age, year	37.8 ± 9.9	42.4 ± 10.5	0.089
Injury type			0.951
LC-I	4	2	
LC-III	6	5	
APC-II	5	6	
APC-III	9	7	
VS	4	5	
CM	3	2	

The posterior pelvic disruptions were reduced anatomically or via open reduction in all 58 patients. The oval track was successfully found and the iliosacral screws were successfully inserted using the axial view projection of the S1 pedicle in all patients. The postoperative pelvic anteroposterior, inlet, and outlet views were made, and the iliosacral screws were found to be well placed along the desired osseous corridor. All the pelvic films were evaluated using the criteria of Van den Bosch *et al*.^13^; no screw perforated a nerve root tunnel, sacral cortex, or spinal canal.

There were no major clinical complications or malpositioned screws. One patient developed a superficial wound infection, which was treated with bedside debridement and further wound care. The respective median times from initial preparation to completion of the first screw insertion in the normal and dysmorphic sacrum groups were 14 ± 5 and 16 ± 4 minutes (*P* = 0.167), and the respective radiation exposure times were 50 ± 9 and 53 ± 12 seconds (*P* = 0.264; Table 2).

**Table 2:** Previous reports regarding operative time per screw and radiation exposure time.

Study and Year	Case No.	Method	Mean Age	Operative Time Per Screw(min)	Radiation Exposure Time(min)
Nork *et al*.^15^	13	Inlet, outlet, lateral view	39	48	126
Tonetti *et al*.^16^	30	Inlet, outlet, lateral view	34.7	35	62
	4	Computer asisted group	48.5	50	21
Mosheiff *et al*.^17^	29	Computerized navigation	16–66	10~15(system preparatory time)	—
Peng *et al*.^4^	18	One C-arm(n = 10)	28.5	45	342
		Two C-arm(n = 8)	31	16	270
Zwingmann *et al*.^18^	24	Navigation system	35 ± 23	72 ± 16	63 ± 15
	32	Inlet, outlet, lateral view	46 ± 20	69 ± 38	141 ± 69
Gras *et al*.^19^	44	2D navigation system	—	62 ± 4	123 ± 12
Kadir *et al*.^20^	7	Inlet, outlet, lateral view	31	—	138
	10	Sacral pedicles view	30	—	52
Our study					
Normal sacrum group	31	Sacral pedicles axial view	37.8 ± 9.9	14 ± 5	50 ± 9
Dysmorphic sacrum group	27		42.4 ± 10.5	16 ± 4	53 ± 12

## Discussion

In the present study, 47% of patients had sacral dysmorphism. All patients in both the normal and dysmorphic sacrum groups had iliosacral screws successfully inserted under the guidance of the axial view projection of the S1 pedicle. There was no significant difference between groups in operating time per screw and radiation exposure time, although both parameters were reduced compared with previous studies^4, 15–20^. There were no severe complications or malpositioned screws in our series.

In our series, iliosacral screws were inserted under the guidance of the axial view projection of the S1 pedicle in both groups through the ilium, across the sacroiliac articulation, and into the superior sacral vertebral bodies using percutaneous techniques. The osseous volume used for iliosacral screw placement is bounded by a narrow corridor of the sacral pedicle, which is the foraminal region of the sacrum and the narrowest part of the iliosacral screw placement^21–24^. Under tangential projection by the sacral pedicle axial view, the foraminal region of the sacrum produces a radiodense oval track imaging, which is the narrow zone of the sacral pedicle surrounded by the sacral ala, vertebral canal, and L5 and S1 nerves. The traditional fluoroscopic projected views used for guiding screw insertion are the inlet, outlet and lateral views. With the traditional fluoroscopic projection technique, surgeons must understand well the correlation between the anatomic landmarks of the posterior pelvis and the corresponding fluoroscopic images, which requires a long learning curve.

In cases where the lumbosacral junction and sacral dysmorphism happened (Fig. 2A,B)^25^, it is difficult to guide screw insertion using the inlet, outlet and true lateral views. Regarding variations in the morphology of the upper sacral segments, the sacral pedicle of S1 (the foraminal region of the sacrum) is always stable and connects and transmits weight from the spine to the ilium, and the direction of the projection only requires minor adjusting to find it. Although in some obese patients the imaging was fuzzy, the sacral pedicle axial view could still be used to find the required region by increasing the C-arm radiation dose.

The operative parameters of seven previous reports were compared with this study and showed in Table 2. Mosheiff *et al*.^17^ and Peng *et al*.^4^, respectively, using the computerized fluoroscopic navigation and two C-arm to implant the sacroiliac screw, and got a shorter operative time. However, the computerized fluoroscopic navigation is more expensive, and not every hospital has the economic capacity to equip it. Two C-arm will take up more operating room space and require more medical staff to cooperate. Kadir *et al*.^20^ applied the axial view projection of S1 pedicle and “sacral mapping” technique (two orthogonal subcutaneous K-wires) to treat ten pelvic posterior ring disruption patients. The control group consisted of seven patients. In these, the screw was placed with the use of the conventional projections (inlet/outlet and lateral). The results showed that this combined technology could significantly reduce the radiation time and dose, which confirmed the efficacy of axial view projection of S1 pedicle.

During traditional positioning of the iliosacral screw, inlet, outlet and lateral views are used for guidance^1^, and safe screw placement is dependent on accurate identification of the critical boundaries of the volume of bone fluoroscopically. The starting point and insertion direction of the guide pin must be determined using the three views, and any adjustment of the direction of the guide pin needs to be repeated on the three views to obtain the ideal target point and intra-osseous trajectory. The surgeon must therefore spend more time and withstand substantial radiation exposure while changing all three views throughout the procedure^5, 6, 26^. Any changes in the direction of the guide pin must also be confirmed on the inlet and outlet views. When familiar with the axial view of the pedicle of S1, the surgeon can rapidly obtain the oval track image that demonstrates the correct pathway of the guide pin in only one view. Once obtained, the axial view provides immediate visual feedback regarding the pathway of the guide pin, which minimizes the amount of potentially dangerous radiation exposure, and markedly decreases the operating time.

Safe placement of iliosacral screws is challenging. The insertion technique must be precise to avoid injury to the lumbosacral nerve trunk, cauda equina roots, and S1 nerve. Some experts report that the optimal angle of the inlet and outlet views is when some anatomic landmarks become radiodense^27^. Recently, CT fluoroscopy and computer-assisted navigation have been evaluated to improve the accuracy of screw positioning, which is available in most institutions^28, 29^. Moreover, screw malpositioning with injury to local neurovascular structures reportedly occurs in 2–15% of cases, even by experienced surgeons^6, 9, 10^. Using the new technique in the present study, the appearance of a radiodense oval track imaging (the axial view of the pedicle of S1) on axial view indicated that the guide pin was located inside the oval track. The guide pin will not penetrate the cortical bone, which avoids injury to the lumbosacral nerve trunk, cauda equina roots, and S1 nerve. Furthermore, adjusting the direction of the guide pin is easier to accomplish outside the bone than inside the bone.

The main limitation of our study is that this new projection technique needs a certain learning curve to find the axial view of the S1 pedicle^30^. However, compared with the traditional fluoroscopic projected technique including inlet, outlet and lateral views, this procedure is easier to understand and achieve, even for less experienced surgeons. If the lumbosacral junction and sacral dysmorphism happened, the radiodense oval track could also be found by this projection technology. Another study limitation is the lack of biomechanical comparison with traditional fixed methods^31, 32^.

In summary, preoperative pelvic CT is necessary to determine the sacral osseous anatomy, bone quality and osseous injury. Axial view projection of the S1 pedicle is an optimal radiographic technique for iliosacral screw placement in both the normal and dysmorphic sacrum, and results in less radiation exposure and complications than the conventional method.
